# Increased CHST15 follows decline in arylsulfatase B (ARSB) and disinhibition of non-canonical WNT signaling: potential impact on epithelial and mesenchymal identity

**DOI:** 10.18632/oncotarget.27634

**Published:** 2020-06-16

**Authors:** Sumit Bhattacharyya, Leo Feferman, Xiaorui Han, Ke Xia, Fuming Zhang, Robert J. Linhardt, Joanne K. Tobacman

**Affiliations:** ^1^ Department of Medicine, University of Illinois at Chicago, Chicago, IL, USA; ^2^ Jesse Brown VAMC, Chicago, IL, USA; ^3^ Department of Chemistry and Chemical Biology Rensselaer Polytechnic Insitute, Troy, NY, USA

**Keywords:** sulfotransferase, sulfatase, chondroitin sulfate, Wnt, EMT

## Abstract

Expression of CHST15 (carbohydrate sulfotransferase 15; chondroitin 4-sulfate-6-sulfotransferase; BRAG), the sulfotransferase enzyme that adds 6-sulfate to chondroitin 4-sulfate (C4S) to make chondroitin 4,6-disulfate (chondroitin sulfate E, CSE), was increased in malignant prostate epithelium obtained by laser capture microdissection and following arylsulfatase B (ARSB; N-acetylgalactosamine-4-sulfatase) silencing in human prostate epithelial cells. Experiments in normal and malignant human prostate epithelial and stromal cells and tissues, in HepG2 cells, and in the ARSB-null mouse were performed to determine the pathway by which CHST15 expression is up-regulated when ARSB expression is reduced. Effects of Wnt-containing prostate stromal cell spent media and selective inhibitors of WNT, JNK, p38, SHP2, β-catenin, Rho, and Rac-1 signaling pathways were determined. Activation of WNT signaling followed declines in ARSB and Dickkopf WNT Signaling Pathway Inhibitor (DKK)3 and was required for increased CHST15 expression. The increase in expression of CHST15 followed activation of non-canonical WNT signaling and involved Wnt3A, Rac-1 GTPase, phospho-p38 MAPK, and nuclear DNA-bound GATA-3. Inhibition of JNK, Sp1, β-catenin nuclear translocation, or Rho kinase had no effect. Consistent with higher expression of CHST15 in prostate epithelium, disaccharide analysis showed higher levels of CSE and chondroitin 6-sulfate (C6S) disaccharides in prostate epithelial cells. In contrast, chondroitin 4-sulfate (C4S) disaccharides were greater in prostate stromal cells. CSE may contribute to increased C4S in malignant epithelium when GALNS (N-aceytylgalactosamine-6-sulfate sulfatase) is increased and ARSB is reduced. These effects increase chondroitin 4-sulfates and reduce chondroitin 6-sulfates, consistent with enhanced stromal characteristics and epithelial-mesenchymal transition.

## INTRODUCTION

Carbohydrate sulfotransferase 15 [CHST15; *N*-acetylgalactosamine 4-sulfate 6-*O*-sulfotransferase; GalNAc4S-6ST; also known as BRAG (B-cell Recombination Activating Gene)] is required for the synthesis of chondroitin sulfate E (CSE; [GlcA-GalNAc-4S,6S]_n_, where S corresponds to sulfate) from chondroitin 4-sulfate (C4S; [GlcA-GalNAc-4S]_n_). CSE is composed of alternating β-1,4- and β-1,3- linked D-glucuronate and D-*N*-acetylgalactosamine-4S,6S residues, and is found throughout human tissues. Increases in CHST15 or in CSE, have been recognized in malignant cells and tissues and in models of tumor progression, including in pancreas, ovary, colon, breast, lung, and glioblastoma [1–11]. Reports have shown a direct association between CHST15 and the proliferation of human pancreatic ductal adenocarcinoma cell lines *in vivo* and *in vitro* [1–3]. The time to recurrence was shorter and survival was less in the group with higher CHST15 expression compared with the negative-to-moderate CHST15 expression group. CHST15 was highly expressed in unfavorable ovarian cancers and was associated with worse prognosis [4–7]. In a model of glioblastoma, inhibition of increased matrix sulfation, attributable to increased CSE and increased chondroitin 4-sulfate, reduced invasiveness [11]. Increases in CHST15 have also been associated with increased fibrosis in cardiac, pulmonary, esophageal, and colonic tissues [12–15].

In our previous studies, we demonstrated functional effects due to the increase in chondroitin 4-sulfate (C4S), which follows decline in arylsulfatase B (ARSB, *N*-acetylgalactosamine-4-sulfatase) [16–23]. ARSB is the enzyme that removes 4-sulfate groups from *N*-acetylgalactosamine 4-sulfate, and is required for the degradation of C4S and dermatan sulfate (DS; [IdoA-GalNAc-4S]_n_) [24–26]. These effects were attributed to the impact of increased C4S on binding with important mediators of cell functions, including galectin-3 and SHP2 (PTPN11), the ubiquitous non-receptor tyrosine phosphatase. When C4S was increased, due to inhibition of ARSB, binding of galectin-3 with C4S declined and binding of SHP2 with C4S increased [16–23], leading to significant effects on transcriptional and signaling events.

Chondroitin sulfates, including chondroitin 4-sulfate (C4S), chondroitin 6-sulfate (C6S; [GlcA-GalNAc-6S]_n_), chondroitin-2,4-sulfate (CSD; [GlcA-2S-GalNAc-4S]_n_), and CSE, are important components of cells and the extracellular matrix (ECM) in all human tissues and throughout living organisms. Biosynthesis of CSE by CHST15 proceeds by transfer of the sulfate residue from 3′-phosphoadenosine-5′-phosphosulfate (PAPS) to the 6-OH of GalNAc4S of C4S [27]. A mechanism of CHST15 expression has not been reported previously.

In our published reports, marked decline in arylsulfatase B (ARSB; *N*-acetylgalactosamine-4-sulfatase) was associated with earlier biochemical recurrences and with higher Gleason scores in prostate cancers [28, 29]. These effects were attributed to signaling mechanisms resulting from the increased sulfation of C4S when ARSB was reduced. Also, in malignant prostate tissues, ARSB activity and expression were reduced and activity and expression of GALNS (*N*-acetylgalactosamine-6-sulfatase) were increased [16–19]. Consistent with these findings, marked increases in C4S, decline in C6S, and increased C4S: C6S ratio were shown in malignant prostate tissues and in human prostate stem cells [17, 19]. In tissue obtained by laser capture microdissection, ARSB expression was markedly reduced in malignant prostate epithelium, but not in stroma, and expression of GALNS was greater in malignant epithelium than in normal epithelium and much greater in normal epithelium than in normal or malignant stroma [19]. In contrast, ARSB expression was significantly greater in normal or malignant prostate stromal tissue than in epithelium. Expression of CHST15 and production of CSE are of particular interest in this context. Since chondroitin-6 sulfatase can remove the 6-sulfate group of CSE [30–33], epithelial C4S content can be increased by this process, modifying the relative content of C4S to C6S. This may contribute to EMT in the epithelium, since C4S is relatively greater in stroma. In this report, we present data about expression of chondroitin sulfotransferases, chondroitin disaccharides, and a non-canonical WNT-pathway for regulation of expression of CHST15 in prostate epithelium. The pathway derives from prior work which detailed the activation of WNT signaling in prostate epithelium and stem cells following decline of ARSB and the resulting increase in C4S [16–19]. SHP2 was inhibited by the increase in chondroitin 4-sulfate; phospho-ERK1/2 activation was sustained; c-Myc nuclear localization was increased; expression of DNA methyltransferases was increased; and methylation of the DKK3 promoter was increased. Since DKK3 acts to inhibit Wnt signaling, reduced expression of DKK3 disinhibited Wnt signaling, leading to activation of canonical Wnt signaling with enhanced nuclear localization of β-catenin and associated transcriptional events.

The experiments presented in this report demonstrate activation of non-canonical Wnt signaling, leading to the enhanced expression of CHST15 through activation of Rac-1 GTPase and phospho-p38 MAPK, with effects on GATA-3. This pathway is consistent with reports in the literature which indicate the effects of Wnt3A on Rac-1 GTPase [34, 35], the activation of phospho-p38 MAPK by Rac-1 GTPase [36–40], the phosphorylation and nuclear translocation of GATA-3 by phospho-p38 MAPK [41–45], and increased CHST15/BRAG expression following binding of GATA-3 to the CHST15/BRAG promoter [46–49]. We have addressed these interactions in prostate epithelial cells stimulated by Wnt3A-containing spent media from prostate stromal cells. Experiments to elucidate the mechanism of regulation of CHST15 expression in prostate epithelium are presented in this report.

## RESULTS

### Characteristics of stromal and epithelial normal and malignant tissue and normal prostate stromal and epithelial cells

Previously, we have presented images from laser-capture microdissection (LCM) of prostate epithelial and stromal tissues obtained from prostate adenocarcinomas and adjacent non-malignant tissue [19]. The prostate tissues were obtained from de-identified, previously untreated subjects in their late 50s with cancers staged as T2c or T3a, with no evidence of nodal or metastatic involvement, who underwent prostatectomy for prostate cancer detected on biopsy. The tissues showed negligible E-cadherin mRNA expression in the stroma and significantly less in the malignant epithelium than in the normal epithelium. Vimentin mRNA expression was negligible in the epithelium and significantly higher in both normal and malignant stroma. Expression of Cyclin D1, c-Myc, Axin, and NKD1 were higher in malignant epithelial tissue.

Previously, the activity of ARSB was reported to be higher in normal stromal cells, compared to normal epithelial cells (140 ± 8 vs. 111 ± 7 nmol/mg protein/h) [16]. This is consistent with findings that ARSB mRNA expression was higher in prostate stroma, both benign and malignant, than in prostate epithelium obtained by LCM [19]. ARSB expression was lowest in the malignant epithelium, where the GALNS expression was highest [19]. Also, ARSB activity was reduced by ~46% in the malignant vs. the normal prostate tissue, and GALNS activity was increased by 20% in the malignant tissue [17]. Consistent with these findings, the ratio of chondroitin 4-sulfate to chondroitin 6-sulfate [C4S: C6S] was higher in malignant prostate tissue than in normal tissue by almost three-fold [17].

### CHST 15 expression increased in malignant prostate epithelium and in prostate epithelial cells following ARSB knockdown

**Figure 1:**
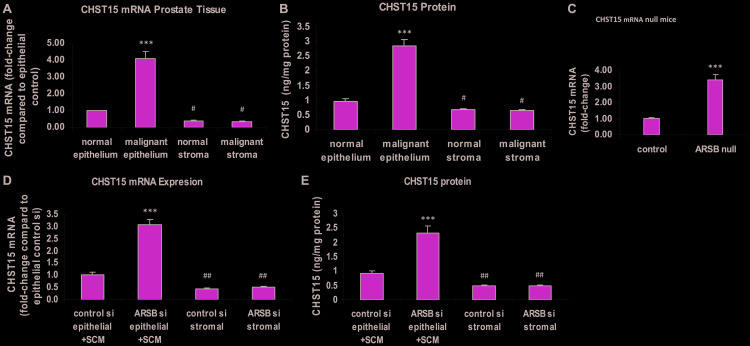
Chondroitin sulfotransferase (CHST) 15 (chondroitin 4-sulfate 6-O-sulfotransferase) is increased in malignant prostate epithelial tissue, in prostate tissue of ARSB-null mice, and in prostate epithelial cells when ARSB is reduced. (**A**) In laser-microdissected normal and malignant human prostate epithelium and stroma, CHST15 mRNA expression is increased in the malignant epithelial tissue compared to normal epithelial tissue (*p* < 0.001; *n* = 6). In the normal and malignant stroma, CHST15 expression is less than in the normal epithelium (*p* < 0.05; *n* = 6). (**B**) In the laser-microdissected prostate tissues, CHST15 protein detected by ELISA was significantly greater in the malignant prostate tissue (*p* < 0.001; *n* = 3). Stromal values are significantly less than in the normal epithelial tissue (*p* < 0.05; *n* = 3). (**C**) In prostate tissues from ARSB-null mice (Strain 005598, Jackson Labs), the CHST15 mRNA was significantly more than in the prostate tissue from normal C57BL/6J controls (*p* < 0.001; *n* = 6). (**D**) In cultured human prostate epithelial cells (CRL-2850, ATCC) treated with prostate stromal cell (CRL-2854, ATCC) spent media in 1:1 ratio with epithelial cell media, CHST15 expression increased following ARSB silencing by siRNA in the epithelial cells (*p* < 0.001; *n* = 6). Expression was significantly higher in the epithelial cells treated with control siRNA than in the stromal cells (*p* < 0.01; *n* = 6). (**E**) Correspondingly, the CHST15 protein determined by ELISA was significantly greater in the epithelial cells grown with spent media from the stromal cells in 1:1 combination with epithelial cell media and ARSB silencing by siRNA (*p* < 0.001; *n* = 3). [ARSB = arylsulfatase B; CHST = chondroitin sulfotransferase; SCM = prostate stromal cell spent media; si = siRNA; ^***^ for *p* < 0.001 greater than control; ^##^ for *p* < 0.01 and ^#^ for *p* < 0.05 less than control]

Carbohydrate sulfotransferase 15 (CHST15; *N*-acetylgalactosamine 4-sulfate 6-*O* **-**sulfotransferase; GalNAc4S-6ST) is the sulfotransferase which adds 6-sulfate to *N*-acetylgalactosamine 4-sulfate residues of C4S to form chondroitin sulfate E. CHST15 expression was determined in malignant and adjacent normal epithelium and stroma of human prostate tissues obtained by laser capture microdissection (LCM). Total RNA was purified from the samples and subjected to RT-PCR with CHST15 specific primers. CHST15 expression in the malignant epithelium was 4.1 ± 0.4 times the level in the normal prostate epithelium (*p* < 0.001) (Figure 1A). The corresponding CHST15 protein was 2.9 ± 0.2 ng/mg protein in the malignant epithelium, compared to 1.0 ± 0.1 ng/mg protein in the normal epithelium (*p* < 0.001) and ~0.7 ng/mg protein in the normal and malignant stromal tissue (*p* < 0.05) (Figure 1B). In prostate tissue of the ARSB-null mice, the expression of CHST15 was about 3.4 times the level in the prostate of the control mice (*p* < 0.001) (Figure 1C). In cultured prostate epithelial cells (PEC), CHST15 mRNA (Figure 1D) and protein (Figure 1E) increased significantly following exposure to spent prostate stromal cell media (SCM) mixed 1:1 with epithelial cell media and ARSB silencing (*p* < 0.001). The CHST15 expression in the normal epithelial cells was significantly greater than the level in either normal or malignant stromal cells (*p* < 0.01).

### Expression of other chondroitin sulfotransferases

**Figure 2:**
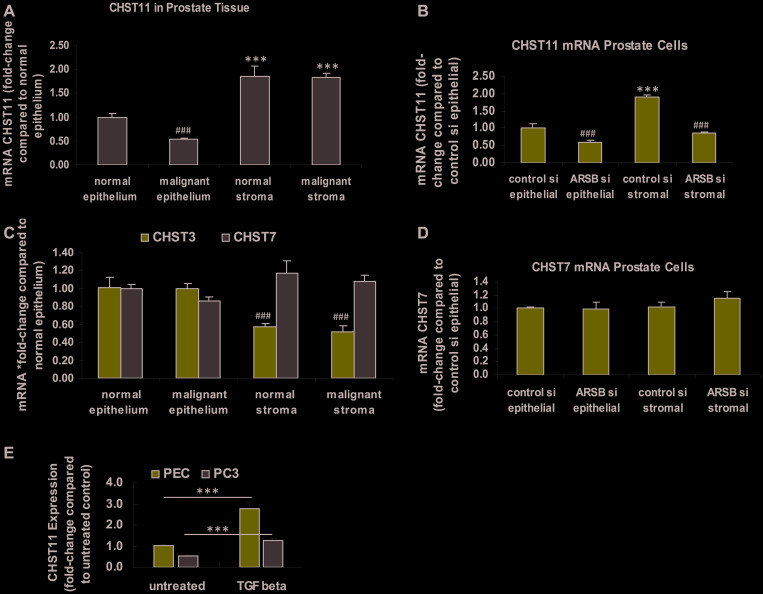
Expression of CHST11, CHST3 and CHST7 in epithelium and stroma. (**A**) In the normal and malignant prostate stromal and epithelial tissues obtained by laser capture -microdissection (LCM), mRNA expression of the chondroitin-4-sulfotransferase CHST11 is significantly greater in the stroma, both normal and malignant, than in the epithelium (*p* < 0.001, *n* = 6). CHST expression is significantly lower in the malignant epithelium, compared to the normal epithelium (*p* < 0.001; *n* = 6). (**B**) In cultured normal prostate stromal cells, CHST11 expression is significantly more than in epithelial cells (*p* < 0.001; *n* = 6). ARSB silencing by siRNA leads to declines in CHST11 expression in both stromal and epithelial cells (*p* < 0.001; *n* = 6). (**C**) In the normal and malignant prostate stromal and epithelial tissues obtained by LCM, the mRNA expression of CHST3, a chondroitin-6-sulfotransferase, is higher in the epithelium than in the stroma, and similar in normal and malignant tissue (*p* < 0.001, *n* = 6). CHST7, another chondroitin 6-sulfotransferase, is not significantly different in stroma vs. epithelium or in malignant vs. normal tissue. (**D**) There are no significant differences in CHST7 expression between the prostate stromal and epithelial cells when ARSB is silenced. (**E**) The expression of CHST11 is increased in both the normal human prostate epithelial cells (CRL-2850) and in the malignant PC-3 cell line (CRL-1435, ATCC) following exposure to exogenous TGF-β (10 ng/ml × 24 h; *p* < 0.001, *n* = 3). This is consistent with previous data about the effect of TGF-β on CHST11 expression and shows that the pathway for expression is intact in the epithelial cells [50]. ^***^ represents *p* ≤ 0.001 and greater than control; ^###^ represents *p* ≤ 0.001 and less than control. [ARSB = arylsulfatase B; CHST = chondroitin sulfotransferase; LCM = laser-capture microdissection; PC-3 = metastatic prostate cell line; PEC = prostate epithelial cell; si = siRNA; TGF-β = transforming growth factor β]

In contrast to the observed increase in CHST15 expression, the expression of CHST11 was significantly reduced in the malignant prostate epithelium and lower in the normal epithelium than in either normal or malignant stroma (*p* < 0.001) (Figure 2A). In prostate epithelial and stromal cells, the CHST11 expression declined when ARSB was silenced (*p* < 0.001) (Figure 2B). Expression of CHST3, a chondroitin-6-sulfotransferase, was significantly less in the normal and malignant stroma than in the epithelium (*p* < 0.001) (Figure 2C). Expression of CHST7, another chondroitin-6-sulfotransferase, was not significantly different in stromal vs. epithelial tissue (Figure 2C). In the prostate epithelial and stromal cells, CHST7 expression was similar with or without ARSB silencing (Figure 2D). As a positive control and based on previous experiments [50], the effect of exposure to exogenous TGF-β was tested. Exogenous TGF-β (10 ng/ml × 24 h) significantly increased the CHST11 expression in both normal human prostate epithelial cells and in malignant PC-3 cells (*p* < 0.001) (Figure 2E).

### Disaccharide analysis supports sulfotransferase data

**Figure 3:**
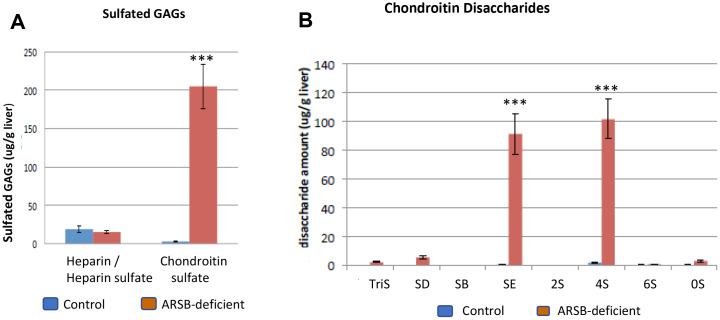
Disaccharide analysis shows increases in chondroitin sulfate E and chondroitin 4-sulfate disaccharides in ARSB-null mouse. (**A**) Measures of chondroitin sulfates and heparin/heparan sulfates in hepatic tissue of ARSB-null mice demonstrate marked increase in chondroitin sulfates, compared to control hepatic tissue from C57BL/6J mice (ug/g liver; *n* = 4 per group). (**B**) The distribution of chondroitin sulfate disaccharides in the hepatic tissue of the ARSB-null mice compared with the normal control mice shows marked increase in 4S and SE disaccharides (mg/g liver; *n* = 4 per group). [GAG = glycosaminoglycan; HS = heparin/heparan sulfate; CS = chondroitin sulfate; chondroitin sulfate disaccharides are: 0S = (UA-GalNAc); 4S = (UA-GalNAc4S); 6S = (UAGalNAc6S); 2S = (UA2S-GalNAc); SB = (UA2S-GalNAc4S); SD = (UA2S-GalNAc6S); SE = (UA-GalNAc4S6S); triS = (UA2SGalNAc4S6S); ^***^ for *p* < 0.001].

Disaccharide analysis was performed in tissues from the ARSB-null mouse and in human prostate epithelial and stromal cells. Findings in hepatic tissue of the ARSB-null mouse showed almost a 10-fold increase in chondroitin sulfates, increasing to ~200 μg/g liver, with no increase in heparin/heparan sulfate glycosaminoglycans (*p* < 0.001) (Figure 3A). Increased C4S disaccharides (ΔUA-GalNAc4S, designated 4S) and chondroitin-4,6-disaccharides (ΔUA-GalNAc4S6S, designated SE) accounted for most of the increase (Figure 3B). The quantitative data show an increase from 0.42 ± 0.32 μg/g liver tissue to 91.3 ± 28.2 mg/g in CSE disaccharide (*p* = 0.0052; unpaired *t*-test, two-tailed, unequal SD; *n* = 4) and from 1.87 ± 1.0 μg/g to 102 ± 27.3 μg/g in C4S disaccharide (*p* = 0.0076; unpaired t-test, two-tailed, unequal SD; *n* = 4) in the ARSB-null mouse hepatic tissue (Table 1). Small increases in other disaccharides accounted for less than 12 μg/g total increase.

**Table 1:** Chondroitin sulfate disaccharides in mouse liver [μg/g ± (standard deviation)]

Disaccharides	TriS	SD	SB	SE**	2S	4S**	6S	0S	Total CS μg/g liver
Control (*n* = 4)	0.00 (0.00)	0.03 (0.02)	0.00 (0.00)	0.42 (0.32)	0.00 (0.00)	1.87 (1.0)	0.22 (0.22)	0.22 (0.09)	2.76 (1.32)
ARSB null (*n* = 4)	2.18 (0.77)	5.36 (1.70)	0.24 (0.03)	91.3 (28.2)	0.00 (0.00)	102 (27.3)	0.88 (0.14)	2.95 (2.02)	205 (57.9)

The quantitative disaccharide data (mg/g) show significant differences in the SE and 4S disaccharides (*p* = 0.0052, *p* = 0.0076; unpaired *t*-test, two-tailed, unequal standard deviations, *n* = 4).

^a^Disaccharides are: TriS: ΔUA2SGal-NAc4S6S); SD: ΔUA2S-GalNAc6S; SB: ΔUA2S-GalNAc4S; SE: ΔUA-GalNAc4S6S; 2S: ΔUA2S-GalNAc; 4S: ΔUA-GalNAc4S; 6S: ΔUA-GalNAc6S; 0S: ΔUA-GalNAc where UA = uronic acid; GalNAc = N-acetylgalactosamine; S = sulfate

ARSB = arylsulfatase B, N-acetylgalactosamine-4-sulfatase; CS = chondroitin sulfates

In the prostate stromal cells, the percentage of 4S disaccharide content in relation to the total chondroitin sulfate content is higher than in the prostate epithelial cells (*p* = 0.039; unpaired t-test, two-tailed, equal SD; *n* = 3). In the epithelial cells, the percentages of chondroitin 6-sulfate disaccharides (designated 6S) and SE disaccharides are higher than in the stromal cells (*p* = 0.019, *p* = 0.0002; unpaired *t*-test, two tailed, equal SD; *n* = 3) (Table 2). These disaccharide values are consistent with the CHST3, CHST11, and CHST15 data presented above. Higher C4S disaccharides and CHST11 expression are present in the stromal tissue and in the prostate stromal cells. Higher SE disaccharides and higher CHST15 are present in the epithelial tissue and in the prostate epithelial cells. Higher chondroitin 6-sulfate disaccharide (designated 6S) in the epithelial cells is consistent with higher CHST3 expression in the epithelial tissue.

**Table 2:** Chondroitin sulfate disaccharides % ± (standard deviation)

*Disaccharides^a^*	TriS	SD	SB	SE ^***^	2S	4S ^*^	6S ^*^	0S
Prostate Epithelial Cells	0 (0)	0.2 (0)	0.3 (0.1)	4.2 (0.5)	0.1 (0)	81.5 (0.9)	12.5 (0.3)	1.2 (0.1)
Prostate Stromal Cells	0 (0)	0.5 (0.2)	0.6 (0.1)	0.1 (0.1)	3.0 (0.5)	84.7 (1.6)	9.8 (1.2)	1.3 (0.1)

The percentages of disaccharides in prostate stromal and epithelial cells cultured under routine conditions show increased 4S disaccharides in the stromal cells (84.7 ± 1.6% vs. 81.5 ± 0.9%) and 6S (12.5 ± 0.3% vs. 9.8 ± 1.2%) and SE (4.2 ± 0.5% vs. 0.1 ± 0.1%) disaccharides in the epithelial cells (*p* = 0.039, *p* = 0.019, *p* = 0.0002, respectively, unpaired *t*-test, two-tailed, equal standard deviations, *n* = 3). Increased 2S disaccharides are present in the prostate stromal cells, compared to the epithelial cells, but the percentages are all small (<3.0%).

^a^Disaccharides are: TriS: ΔUA2SGal-NAc4S6S); SD: ΔUA2S-GalNAc6S; SB: ΔUA2S-GalNAc4S; SE: ΔUA-GalNAc4S6S; 2S: ΔUA2S-GalNAc; 4S: ΔUA-GalNAc4S; 6S: ΔUA-GalNAc6S; 0S: ΔUA-GalNAc where UA = uronic acid; GalNAc = N-acetylgalactosamine; S = sulfate

^***^ for *p* < 0.001, ^**^ for *p* < 0.01, and ^*^ for *p* < 0.05 by unpaired *t*-test, two-tailed.

### Wnt signaling, phospho-p38 MAPK, and Rac-1 GTPase required for increased CHST15 expression

**Figure 4:**
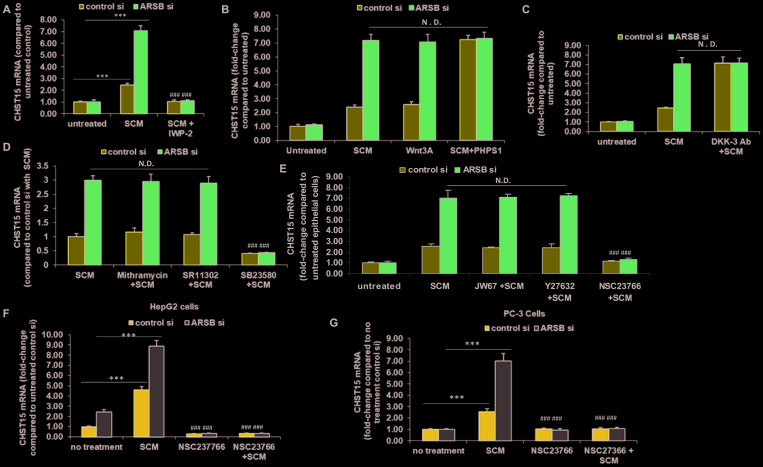
Modification of CHST15 expression in prostate epithelial cells by effects on Wnt signaling. (**A**) Prostate stromal cells were treated with IWP-2 (1 mg/ml × 24 h), an inhibitor of Wnt intracellular processing and secretion. Spent media from the IWP-2-treated stromal cells in 1:1 combination with prostate epithelial cell media blocked the increase in CHST15 expression by the prostate epithelial cells (*p* < 0.001; *n* = 3). When ARSB was silenced by siRNA in the epithelial cells, treatment with the spent cell media (SCM) from the prostate stromal cells (in 1:1 combination with the epithelial cell media), the CHST15 expression was greater (*p* < 0.001; *n* = 3), and was inhibited when the stromal cells were treated by IWP-2. (**B**) CHST15 expression increased in the presence of stromal cell spent media and exposure to exogenous Wnt3A (1 ng/ml × 24 h; *p* < 0.001; *n* = 3). ARSB inhibition by siRNA led to further increases. When epithelial cells were treated with the SHP2 inhibitor PHPS1 (30 μM × 24 h), CHST15 expression increased to ~7.3 times the level in the control silenced cells, but did not increase further when ARSB was silenced. (**C**) When DKK3 capture antibody (0.2 ug/ml × 24 h) was added to the media, CHST15 expression increased in the epithelial cells, due to the disinhibition of Wnt signaling by the antibody (*p* < 0.001; *n* = 3). (**D**) Neither mithramycin (250 nM x 24h), an inhibitor of SP1, nor SR11302 (5 μM × 24 h), an inhibitor of AP-1 DNA binding, inhibited the increase in CHST15 expression from exposure to spent stromal cell media. However, SB23580 (10 μM × 24 h), an inhibitor of phospho-p38 MAPK, blocked the SCM-induced increase in CHST15 expression (*p* < 0.001; *n* = 3). (**E**) JW67 (4 mg/ml × 24 h), an inhibitor of canonical Wnt signaling, and Y27632 (10 μM × 24 h), an inhibitor of Rho kinase, did not reduce the increases induced by SCM and ARSB silencing. However, NSC23766 (1 ng/ml × 24 h), an inhibitor of Rac-1 GTPase, blocked the SCM- and ARSB-induced increases in CHST15, demonstrating a requirement for Rac-1 GTPase (*p* < 0.001; *n* = 3). (**F**) Similarly, in HepG2 cells, NSC23766, the inhibitor of Rac-1 GTPase, blocked the increase in CHST15 expression following treatment with prostate stromal cell spent cell media in 1:1 combination with HepG2 media and following ARSB silencing by siRNA (*p* < 0.001; *n* = 3). (**G**) In PC-3 cells, NSC23766 blocked the increase in CHST15 expression induced by prostate stromal cell spent media and ARSB silencing (*p* < 0.001; *n* = 3). [ARSB = arylsulfatase B; CHST = chondroitin sulfotransferase; DKK = Dickkopf Wnt signaling pathway inhibitor; JW67 = β-catenin nuclear translocation inhibitor; N.D. = no difference; NSC23766 = Rac-1 GTPase inhibitor; PHPS1 = SHP2 inhibitor = phenylhydrazonopyrazolone sulfonate; SB203580 = p38 MAPK inhibitor; SR11302 = AP-1 inhibitor; Y27632 = Rho kinase inhibitor; SCM = stromal cell spent media; si = siRNA]. ^***^ for *p* < 0.001 greater than control; ^###^ for *p* < 0.001 less than control].

Previously, mRNA expression of arylsulfatase B (N-acetylgalactosamine-4-sulfatase) was reported to be markedly less in malignant epithelium than in normal epithelium, and much less in epithelium than in stroma in tissue obtained by LCM [19]. Findings indicated that ARSB regulated Wnt signaling in prostate through effects on SHP2 and DKK3, a Wnt signaling pathway inhibitor. Based on these findings, the impact of ARSB silencing and of Wnt3A exposure on the expression of CHST15 was investigated in the normal epithelial cells. Wnt3A in the spent stromal cell media was previously shown to activate Wnt signaling in the prostate epithelial cells [19]. When the epithelial cells were treated with spent stromal cell medium (SCM) in 1:1 combination with epithelial cell growth media, CHST15 expression increased, and increased more when ARSB was silenced. These effects were blocked when the epithelial cells were exposed to SCM from stromal cells which had been treated with IWP-2 (1 μg/ml), an inhibitor of Wnt processing and secretion (*p* < 0.001, *n* = 3) (Figure 4A). When the stromal cells were treated with exogenous Wnt3A (1 ng/ml), CHST15 expression increased. Treatment with the SHP2 inhibitor PHPS1 (30 μM) further increased the CHST15 (*p* < 0.001, *n* = 3). ARSB silencing enhanced the effects of SCM and Wnt3A, but showed no additive effect with PHPS1, consistent with mediation of the impact of ARSB silencing by inhibition of SHP2 (Figure 4B). When epithelial cells were exposed to media with DKK3 neutralizing antibody, CHST15 expression increased due to enhanced Wnt signaling in the absence of DKK3 (Figure 4C). ARSB silencing had no additional impact, reflecting that the downstream impact of ARSB silencing on Wnt signaling in the epithelial cells is through inhibition of DKK3 expression [19].

To elucidate the signaling cascade following exposure of the epithelial cells to Wnt3A or SCM, the epithelial cells were treated with epithelial cell media and SCM (1:1), either without additive or in combination with inhibitors of specific signaling pathways. The effects of exposure for 24 h to SB23580 (10 μM), a p38 MAPK inhibitor, SR11302 (5 μM), an inhibitor of AP-1 DNA binding, and mithramycin (250 nM), an inhibitor of Sp1 binding to DNA, following control or ARSB silencing were tested on CHST15 expression (Figure 4D). Of these inhibitors, only phospho-p38 MAPK inhibition blocked CHST15 expression (*p* < 0.001). In other experiments, effects of JW67 (4 mg/ml), an inhibitor of Wnt/β-catenin canonical signaling pathway which induces β-catenin destruction, the Rho inhibitor Y-27632 (10 μM), and the Rac-1 GTPase inhibitor NSC-23766 (1 ng/ml) for 24 h following control silencing and ARSB silencing were tested (Figure 4E). The Rac-1 GTPase inhibitor NSC-23766 totally blocked the response to SCM after control or ARSB silencing (*p* < 0.001), whereas neither JW67 nor Y27632 had any impact. These findings indicated that the activation of Rac-1 GTPase affected CHST15 expression, whereas neither the canonical Wnt-β-Catenin pathway nor Rho GTPase had any impact. Similar results, showing inhibition of CHST15 expression by Rac-1 GTPase inhibition were evident in HepG2 cells (Figure 4F) and in PC3 prostate cells (Figure 4G).

### Phospho-p38/p38 MAPK increased by Wnt signaling and Rac-1 GTPase

**Figure 5:**
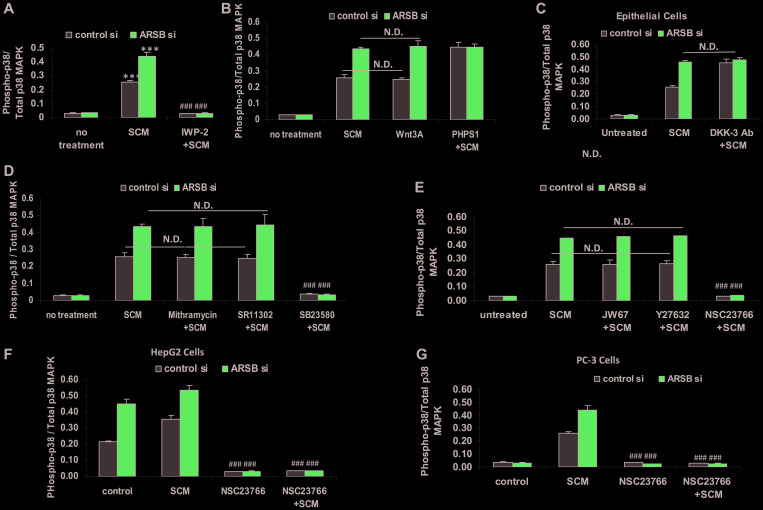
Increase in phospho-p38 / total p38 MAPK follows decline in ARSB and activation of Wnt signaling. (**A**) Exposure of the prostate stromal cells to the Wnt inhibitor IWP-2 blocked the increase in phospho-p38/total p38 MAPK ratio in the epithelial cells, when they were exposed to spent media from the stromal cells (SCM) in 1:1 combination with the epithelial cell media (*p* < 0.001, *n* = 3). (**B**) In the prostate epithelial cells, exposure to prostate stromal cell spent media (SCM; 1:1 with epithelial cell media), Wnt3A (1 ng/ml × 24 h), and PHPS1 (30 μM) × 24 h increased the phospho-p38 / total p38 MAPK ratio (*p* < 0.001; *n* = 3). Effects of exposure to the SCM and Wnt3A at this concentration were similar. (**C**) Addition of DKK-3 capture antibody (0.2 ug/ml × 24 h; AF1118) to epithelial cell media reduced the effect of DKK3 and increased Wnt signaling, leading to increased phospho-p38/total p38 MAPK ratio (*p* < 0.001; *n* = 3). (**D**) Exposure to mithramycin (250 nM x 24 h) or to SR11302 (5 μM × 24 h) had no effect on the increase in phospho-p38/total p38 MAPK. In contrast, SB23580 (10 μM × 24 h) totally blocked the increase (*p* < 0.001; *n* = 3). (**E**) JW67 (4 mg/ml × 24 h), an inhibitor of canonical Wnt signaling, and Y27632 (10 μM × 24 h), an inhibitor of Rho kinase, did not block the increases induced by prostate stromal cell spent media (SCM) and ARSB silencing. However, NSC23766 (10 μM × 24 h), which inhibits Rac-1 GTPase, blocked the increase in phospho-p38/total p38 MAPK, indicating a requirement for Rac-1 GTPase (*p* < 0.001; *n* = 3). (**F**) Similarly, in HepG2 cells, NSC23766, the inhibitor of Rac-1 GTPase, blocked the increase in phospho-p38/total p38 MAPK, which followed treatment with the prostate stromal cell spent media (SCM) and ARSB silencing (*p* < 0.001; *n* = 3). (**G**) Also, in PC-3 cells, NSC23766, the inhibitor of Rac-1 GTPase, blocked the increase in phospho-p38/total p38 MAPK induced by spent cell media (SCM) and ARSB silencing (*p* < 0.001; *n* = 3). [ARSB = arylsulfatase B; CHST = chondroitin sulfotransferase; DKK = Dickkopf Wnt signaling pathway inhibitor; JW67 = β-catenin nuclear translocation inhibitor; N.D. = no difference; NSC23766 = Rac-1 GTPase inhibitor; PHPS1 = SHP2 inhibitor = phenylhydrazonopyrazolone sulfonate; SB203580 = p38 MAPK inhibitor; SR11302 = AP-1 inhibitor; Y27632 = Rho kinase inhibitor; SCM = stromal cell spent media; si = siRNA]. ^***^ for *p* < 0.001 greater than control; ^###^ for *p* < 0.001less than epithelial control].

The pathway by which the ratio of phospho-p38/ total p38 MAPK increased was evaluated in the epithelial cells cultured with stromal cells spent media (SCM) in 1:1 combination with epithelial cell growth media × 24 h. The SCM and exogenous Wnt3A both increased the phospho-p38/total p38 MAPK ratio in the epithelial cells (*p* < 0.001) (Figure 5A, 5B). The Wnt inhibitor IWP-2 blocked the increase in phospho-p38/total p38 MAPK ratio (Figure 5A). The SHP2 inhibitor PHPS1 further enhanced the ratio (*p* < 0.001) (Figure 5B). When ARSB was silenced in the epithelial cells, the effects of SCM and Wnt3A increased, but there was no additional response to PHPS1. This is consistent with mediation of the effect of ARSB silencing through inhibition of SHP2, as previously. DKK3 capture antibody also increased the phospho-p38/total p38 MAPK ratio (*p* < 0.001) (Figure 5C). Treatment by mithramycin or the AP-1 inhibitor SR11302 had no effect on the phospho-p38/total p38 MAPK ratio (Figure 5D). As expected, the phospho-p38 inhibitor SB203580 completely blocked the increase. The epithelial cells were treated with JW67, Rho-associated protein kinase (ROCK) inhibitor Y-27632, and Rac-1 GTPase inhibitor (NSC-23766) for 24h following control silencing and ARSB silencing in combination with spent media from the stromal cells to further examine the mechanism by which phospho-p38 increased (Figure 5E). Only the Rac-1 inhibitor blocked the increase in phospho-p38/total p38 MAPK ratio (*p* < 0.001). Similar impact of Rac-1 GTPase inhibition was evident in HepG2 (Figure 5F) and PC-3 cells (Figure 5G). These data collectively show the involvement of phospho-p38 MAPK and Rac-1 GTPase in enhanced epithelial cell expression of CHST15. The increased CHST15 is mediated by the Wnt3A-containing SCM of the stromal cells and the activation of Wnt signaling in the epithelial cells through the disinhibition of DKK3, which follows ARSB-knockdown induced inhibition of SHP2 by increased chondroitin 4-sulfate.

### Nuclear DNA-bound GATA-3 increased by ARSB silencing, Wnt3A and phospho-p38 MAPK

**Figure 6:**
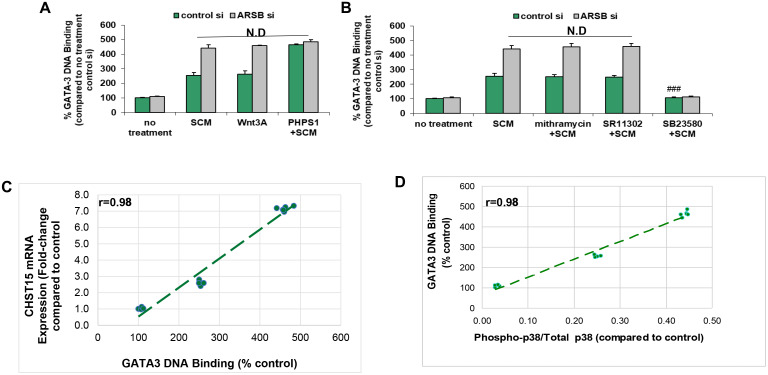
GATA-3 DNA binding is increased by activation of Wnt signaling. (**A**) Exogenous Wnt3A (1 ng/ml × 24 h) had similar effect as the SCM. The SHP2 inhibitor PHPS1 (30 μM × 24 h) further increased the GATA-3 DNA binding (*p* < 0.001; *n* = 3). ARSB silencing produced no additional increase when cells were treated with PHPS1. (**B**) GATA-3 DNA binding was unaffected by mithramycin (250 nM × 24 h) or by SR11302 (5 μM × 24 h). In contrast, the phospho- p38-MAPK inhibitor SB23580 (10 μM × 24 h) blocked the binding, consistent with a requirement for phospho-p38 (*p* < 0.001; *n* = 3). (**C**) The graph shows high correlation (r = 0.98) between CHST15 mRNA expression and GATA-3 nuclear DNA binding. (**D**) The graph shows high correlation (r = 0.98) between GATA-3 nuclear DNA binding and the phospho-p38/total p38 MAPK ratio. [ARSB = arylsulfatase B; CHST = chondroitin sulfotransferase; DKK = Dickkopf Wnt signaling pathway inhibitor; JW67 = β-catenin nuclear translocation inhibitor; N.D. = no difference; NSC23766 = Rac-1 GTPase inhibitor; PHPS1 = SHP2 inhibitor = phenylhydrazonopyrazolone sulfonate; SB203580 = p38 MAPK inhibitor; SR11302 = AP-1 inhibitor; Y27632 = Rho kinase inhibitor; SCM = stromal cell spent media; si = siRNA]. ^***^for *p* < 0.001 greater than control; ^###^ for *p* < 0.001 less than epithelial control].

Since phospho-p38 MAPK has been associated with increased nuclear GATA-3 [41–45], and there is a potential binding site for GATA-3 in the CHST15 promoter [968-979: CGAGATCGCGCC] [46–49], the DNA-bound GATA-3 in the epithelial cells was determined. Stromal cell spent media, ARSB silencing, exogenous Wnt3A and PHPS1, the SHP2 inhibitor, increased the DNA-bound GATA-3 (Figure 6A). The effects of SCM and Wnt3A were enhanced by ARSB siRNA. The stimulatory effect of PHPS1 was not further increased by ARSB knockdown, consistent with the mechanism by which decline in ARSB acts through C4S-mediated SHP2 inhibition. Mithramycin and SR11302 had no effect on the bound nuclear GATA-3, but treatment with SB23580, the phospho-p38 MAPK inhibitor, completely blocked the DNA-bound GATA-3 (*p* < 0.001) (Figure 6B). High correlations (r = 0.98) were identified between CHST15 expression and GATA-3 DNA binding (Figure 6C) and between phospho-p38 / total p38 MAPK ratio and GATA3 DNA binding (Figure 6D).

### Potential for increase in epithelial chondroitin 4-sulfate following increases in CHST15 and GALNS

**Figure 7:**
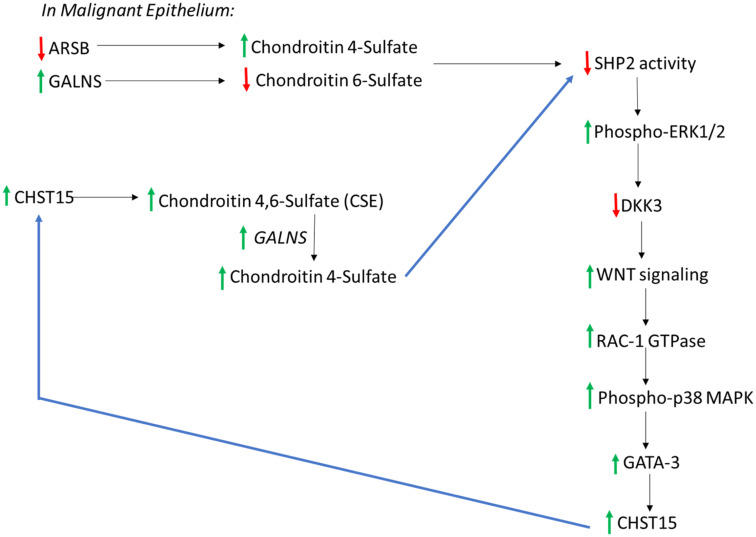
Overall connected pathways of chondroitin 4-sulfate (C4S), chondroitin-4,6-disulfate (CSE), ARSB, and GALNS which may contribute to EMT and progressive increase in SHP2 inhibition. In malignant prostate epithelium, ARSB increased and GALNS declined, in association with increase in CHST15. This pathway may contribute to continuing increases in CHST15 and chondroitin 4-sulfate (C4S), due to inhibition of SHP2 and activation of Wnt signaling through a non-canonical pathway. Noting higher C4S in stroma, with higher C6S in normal epithelium, a relative increase in C4S suggests potential for epithelial to mesenchymal transition in the epithelium. In the normal epithelium, CHST11 expression is less than in stroma and CHST3, leading to C6S, is higher. These findings are consistent with the disaccharide analysis showing lower 4S and higher 6S disaccharides in the prostate epithelial cells. [ARSB = arylsulfatase B; C4S = chondroitin 4-sulfate; C6S = chondroitin 6 = sulfate; CHST = chondroitin sulfotransferase; DKK = Dickkopf Wnt signaling pathway inhibitor; ERK = extracellular regulated kinase; GALNS = N-acetylgalactosamine-6-sulfatase; SHP2 = PTPN11 = non-receptor tyrosine phosphatase 2].

The data are consistent with a cascade in malignant epithelium that links decline in ARSB, increase in C4S, inhibition of SHP2, inhibition of DKK3, disinhibition of Wnt signaling, activation of Rac-1 GTPase and phospho-p38 MAPK, enhanced GATA3 nuclear DNA binding, increased CHST15 expression, and increased production of chondroitin sulfate E (CSE) (Figure 7). In the setting of malignant epithelial cells with high GALNS (chondroitin 6-sulfatase), removal of the 6-sulfate of CSE leads to increased C4S. With low epithelial ARSB, C4S degradation is inhibited, and further increase in C4S may occur. The increase in C4S may lead to further increase in CSE, due to inhibition of SHP2 and iteration of the cascade leading to increased CHST15 and ultimately increased C4S. Since C4S predominates in stroma, the overall increase in C4S suggests a mechanism favoring epithelial-mesenchymal transition in the epithelial cells. In the benign epithelial cells, this process is not anticipated, since ARSB is not reduced and GALNS is not increased from their baseline levels. In the stromal cells, baseline ARSB is higher, so this process is not anticipated to occur.

## DISCUSSION

In prior reports, decline in ARSB and the resultant increase in chondroitin 4-sulfate (C4S) led to activation of Wnt signaling in prostate cells [17–19]. This followed the inhibition of SHP2 (PTP11), a ubiquitous non-receptor tyrosine phosphatase, due to enhanced binding with C4S when ARSB was reduced. Inhibition of SHP2 led to sustained phosphorylations of downstream signaling mediators, including phospho-ERK1/2, leading to enhanced DNA methylation of the DKK (Dickkopf Wnt signaling pathway inhibitor)3 promoter, and disinhibition of Wnt signaling in prostate epithelial and stem cells. Prostate stromal cells produced and secreted Wnt3A, which was needed for the activation of Wnt signaling in the epithelial cells [17]. ARSB expression and activity in the epithelial cells responds to a variety of signals, including androgen and oxygen [19, 25]. Hence, changes in the microenvironment can impact on an extensive array of events by affecting ARSB, including effects on Wnt-mediated transcriptional events through decline in DKK3.

Experiments using tissue obtained by LCM detected higher levels of arylsulfatase B (*N*-acetylgalactosamine-4-sulfatase; ARSB) in prostate stroma than in epithelium, and higher galactose-6-sulfatase (*N*-acetylgalactosamine-6-sulfatase; GALNS) in epithelium than stroma. In the malignant prostate epithelium, GALNS expression was greater and ARSB activity was less than in the benign epithelium [19]. This suggested that SHP2 activity declined as ARSB activity declined and C4S increased in the malignant epithelium. Inhibition of SHP2 has profound impact on multiple downstream signaling events, including the activation of Wnt signaling through effects on DKK3. GALNS by removing 6-sulfate groups from CSE could potentially increase the amount of C4S in the epithelium. Hence, ARSB may act as a tumor suppressor and GALNS as a proto-oncogene due to their impact on chondroitin sulfation and inhibition of SHP2. Effects of SHP2 inhibition are relayed through sustained phosphorylation of a variety of signaling enzymes, including phospho-ERK1/2 and phospho-JNK [17–19].

The disaccharide analysis of hepatic tissue from ARSB-null mice demonstrated marked increase in C4S and CSE disaccharides, from baseline of 2.3 μg/g tissue to over 193 μg/g tissue. The mechanism for the increase in CSE disaccharides was unknown. In this report, we have presented evidence for the mechanism by which expression of CHST15 (chondroitin-4,6-sulfotransferase), which produces CSE, is increased when ARSB is reduced. Study findings present a cascade involving Wnt activation which leads to increased CHST15 expression, mediated by Rac-1 GTPase and phospho-p38 MAPK. Inhibition of canonical β-catenin signaling by JW67 did not block the increase in CHST15 expression, suggesting that non-canonical Wnt signaling is engaged in the mechanism to increase CHST15. Potentially other mechanisms by which Rac-1 GTPase is activated may also be involved in modulation of CHST15 expression. Additional experiments with exogenous Wnt3A would be helpful to confirm direct effects of Wnt3A on the mechanism of CHST15 expression. The effects of the stromal cell media replicated the effects of exogenous Wnt3A (Figures 4B, 5B, 6B), and the Wnt-inhibitor IWP-2 blocked the effects of the stromal cell media in CHST15 expression and phospho-p38/total p38 ratio (Figures 4A, 5A). Future work, such as demonstration of the effects of Wnt3A on phosphorylation of Ror2, will help to clarify the proposed pathway. Also, further assessment of interactions, such as by silencing of both ARSB and GALNS, may help to quantify the effects on SHP2 activity and the relative contributions of each sulfatase to SHP2 inhibition.

Higher CSE and 6S disaccharides are present in the prostate epithelial cells than in the prostate stromal cells, and 4S disaccharides are higher in the prostate stromal cells. Additional findings show lower 2S, SB, and SD disaccharides in the prostate epithelial cells than in the stromal cells, indicating higher levels of 2-sulfated chondroitins in the stromal cells. However, the much higher concentrations of C4S and C6S demonstrate the dominance of these chondroitins, with predominance of C4S in the stromal cells and C6S in the epithelial cells. Additional studies of iduronate-2-sulfatase may help to explain the observed differences in 2-sulfated disaccharides. The disaccharide data are consistent with the results of CHST15, CHST11, and CHST3 expression data in the normal and malignant prostate epithelial and stromal tissues obtained by LCM. Since ARSB expression is less in epithelial tissue than in stromal tissue, and GALNS expression is greater in epithelial than stromal tissue, these enzymes may have substantial impact on epithelial and mesenchymal identity. Changes in these enzymes and in the abundance of the chondroitin sulfates which they degrade may influence epithelial to mesenchymal transition and contribute to malignant transformation.

## MATERIALS AND METHODS

### Cell and tissue samples

Human prostate stromal cells (ATCC^®^: CRL-2854^™^) and prostate epithelial cells (ATCC^®^: CRL-2850^™^) were obtained (ATCC, Manassas, VA) and grown under the recommended conditions, as previously [16]. The human prostate epithelial cells (PEC) were grown in Keratinocyte Serum Free Medium (K-SFM) with 0.05 mg/ml bovine pituitary extract (BPE) and 5 ng/ml epidermal growth factor (EGF). Prostate stromal cells (PSC) were grown in DMEM with 5% fetal calf serum. Cells were maintained at 37° C in a humidified, 5% CO_2_ environment with replenishment of media every third day. Confluent cells in T-25 flasks were harvested by EDTA-trypsin, and sub-cultured in multiwell tissue culture plates under similar conditions. Cells were grown to ~70–80% confluency, treated and harvested by scraping or trypsinization. Spent media from the prostate stromal cells were collected and frozen for future use.

The HepG2 cell line (HB-8065) was obtained (ATCC) and grown in minimum essential medium (MEM) with 10% FBS at 37° C in a humidified, 5% CO_2_ environment, with exchange of media every 2–3 days [21]. The PC3 cell line (CRL-1435), a metastatic prostate cell line, was obtained (ATCC) and grown in F-12K Medium (ATCC) with 10% FBS (Gibco, Millipore Sigma, St. Louis, MO). Cell lysates and nuclear extracts were prepared, as previously described [19]. Some flasks of prostate epithelial and PC-3 cells were treated with TGF-β (10 ng/ml, R&D Systems) for 24 h.

Fresh frozen tissues from radical prostatectomies performed for prostate cancer were obtained from the University of Illinois at Chicago (UIC) Biorepository by an exempt protocol approved by the Institutional Review Board (UIC IRB 2016-0759). Frozen sections were performed and benign and malignant foci of adenocarcinoma, consisting of epithelium and stroma, were identified, isolated, dissected out, and frozen for subsequent analysis, as previously [16, 29]. Tissue samples were from de-identified, previously untreated patients in their late 50s with cancers staged as T2c or T3a, with no evidence of nodal or metastatic involvement, who underwent prostatectomy for prostate cancer detected on biopsy.

Heterozygous arylsulfatase B deficient mice were obtained (Strain 005598; Jackson Laboratories, Bar Harbor, Maine) and bred [16]. Genotyping was performed to detect homozygous or heterozygous mutation or wild-type ARSB. The mutation in ARSB (NM_009712.3) is a G to T mutation at residue 94,560,057 (UCSC database) in exon 2, leading to truncation and reduced activity. Tissues were excised from male ARSB-null mice and control, age-matched C57BL/6J mice at the time of sacrifice, as previously detailed [16]. All animal procedures were approved by the Animal Care Committee of UIC (14-022). ARSB-defective mice and age- and gender-matched C57BL/6J control mice were euthanized by carbon dioxide inhalation and cervical dislocation, and tissues were isolated, excised, and promptly frozen at –80° C. Chst15 expression and chondroitin disaccharides were determined in tissue from the ARSB-null mice.

### Measurement of ARSB and GALNS activity

ARSB activity in the control and treated prostate cells and tissue was determined as reported [24]. Briefly, cells were harvested, and cell homogenates prepared. ARSB activity in the samples was determined in 0.05 M acetate buffer, pH 5.6, containing barium acetate (20 mmol/l:1.0 mol/l Na acetate), using a standard curve of known concentrations of methylumbelliferyl. ARSB activity was expressed as nmol/mg protein/h. GALNS assay was performed with 5 μl cell homogenates made in ddH2O. GALNS activity was determined using 10 mM 4-methylumbelliferyl-β-D-galactoside-6-sulfate, ammonium (MU-βGal-6S) as substrate. Fluorescence readings were taken at 360 nm and 465 nm. GALNS activity was expressed as nmol/mg protein/h.

### Laser capture microdissection (LCM)

Normal and malignant prostate tissue samples were obtained at the time of radical prostatectomy for prostate cancer, frozen. and sectioned in a cryostat. Seven-micron sections were put on polyethylene naphthalate (PEN) membrane slides (Leica Microsystems, Buffalo Grove, IL) [19]. The sections were visualized and cut by microscopic laser capture microdissection (LCM) using Leica LMD 6000 laser dissection microscope and LMD software version 6.5 (Leica Microsystems) and collected in LCM-suitable collection vials (Thermo Fisher Scientific, Waltham, MA).

### QRT-PCR

Total RNA was prepared from malignant and non-malignant prostate tissues and control/ treated cells using an RNeasy Mini Kit (Qiagen). Equal amounts of purified RNAs from the control and treated cells were reverse-transcribed and amplified using Brilliant SYBR Green QRT-PCR Master Mix (Stratagene). Human β-actin was used as an internal control. QRT-PCR was performed using the following specific primers [51, 52]: ARSB (NM_000046) Forward: 5′-AGACTTTGGCAGGGGGTAAT-3′ and Reverse: 5′-CAGCCAGTCAGAGATGTGGA-3′; GALNS (NM_000512) Forward: 5′-ACGGATTTGATGAGTGGTTTG-3′ and Reverse: 5′-GTAGAGGAAAAAGGGGTGGTG-3′; CHST3 (NM_004273) Forward: 5′-GTCTGTCTGGCAATGGAAGAA-3′ Reverse: 5′-CTCAAGCAATCCTCCCACCTT-3′; CHST7 (NM_019886.3) Forward: 5′-GCGAACTCTTTAACCAGGACC- 3′ Reverse: 5′ATGACCTTGTTAGTCCGGCAG-3′; CHST11 (NM_018143) Forward: 5′-GTTGGCAGAAGAAGCAGAGG-3′ Reverse: 5′-GACATAGAGGAGGGCAAGGA-3′; CHST15 human (NM_015892) Forward: 5′-ACTGAAGGGAACGAAAACTGG-3′ Reverse: 5′-CCGTAATGGAAAGGTGATGAG-3′; Chst15 mouse (NM_029935) Forward: 5′-TGTAGCCTGGTCTTTGGATTG-3′ Reverse: 5′-TTTCACATCACTGGGGTTCTC-3′; Chst11 mouse (NM_021439} Forward: 5′-GCTGGAAGTGATGAGGATGAA-3′ Reverse: 5′- CAGGATGGCAGTGTTGGATAG-3′.

Cycle threshold (Ct) was determined during the exponential phase of amplification, as previously [24]. Fold changes in expression were determined from the differences between the Ct values using the formulae: Fold change = 2Δ_3_ with Δ_3_ = Δ_1_-Δ_2_; and Δ_1_ = Ct _control target gene_ – Ct _control β-actin_ and Δ_2_ = Ct _treated target gene_ – Ct _treated β-actin_.

### ARSB silencing by siRNA

Specific siRNAs for ARSB and control siRNAs were procured (Qiagen, Germantown, MD) and used, as previously [16]. Briefly, cells were grown to 70% confluency in 12-well tissue culture clusters, and the medium of the growing cells was aspirated and replaced with 1.1 ml of fresh medium with serum. 0.3 μl of 20 μM siRNA (75 ng) was mixed with 100 μl of serum-free medium and 6 μl of HiPerfect Transfection Reagent (Qiagen). The mixture was incubated at room temperature for 10 min to allow the formation of transfection complexes, and then added dropwise onto the cells. The plate was swirled gently, and treated cells were incubated at 37° C in humidified 5% CO_2_ environment. After 24 h, the medium was exchanged with fresh growth medium. Efficacy of the silencing procedure was determined by measurement of ARSB activity. Activity declined from 140.4 ± 8.4 nmol/mg protein/h to 21.4 ± 0.9 nmol/mg protein/h in the stromal cells and from 110.6 ± 6.8 nmol/mg protein/h to 8.8 ± 0.8 nmol/mg protein/h in the epithelial cells following ARSB silencing by siRNA. Control silencing produced no change in activity. Differences were highly significant (*p* < 0.001, one-way ANOVA).

### Determination of cellular CHST15 protein

CHST15 protein was measured in control and treated prostate epithelial cell extracts by a commercial ELISA (MyBioSource 2600006, San Diego, CA). A microtiter plate was coated with a human CHST15 monoclonal capture antibody. Samples and standards were added to the wells of the microtiter plate. CHST15 protein in the samples was captured by the coated antibody on the plate, and CHST15 protein was detected with biotinylated antibody to CHST15 and streptavidin-HRP. Hydrogen peroxide/tetramethylbenzidine substrate was used to develop the color which was proportional to bound HRP activity. The reaction was stopped, and the optical density of the color in the samples was extrapolated from a standard curve obtained with known standards.

### Phospho-p38/p38 MAPK FACE assay

Commercial, fast-activated cell-based ELISA (FACE; Active Motif, Carlsbad, CA) was used to detect phospho-p38, phosphorylated at Thr180/Tyr182, and total p38, regardless of phosphorylation state, in the prostate epithelial cells. The ratio of phospho-p38 to total p38 was determined following control silencing or silencing of ARSB by siRNA and treatment with stromal cell spent media (SCM) in 1:1 combination with prostate epithelial cell media, and in combination with various inhibitors. Inhibitors include: SB23580, a phospho-p38 MAPK inhibitor (10 μM, EMD Millipore, Billerica, MA); SR11302, an inhibitor of AP-1 DNA binding (5 μM, Calbiochem, Millipore Sigma, Burlington, MA); mithramycin, an inhibitor of Sp1 binding to DNA (250 nM, Sigma-Aldrich, St. Louis, MO); PHPS1, a chemical SHP2 inhibitor (phenylhydrazonopyrazolone sulfonate, 30 μM, Sigma-Aldrich). Prostate epithelial cells were exposed to these inhibitors or to recombinant human Wnt3A (1 ng/ml; R&D Systems, Minneapolis, MN) for 24h post control or ARSB silencing.

### GATA-3 transcription factor assay

Active GATA-3 binding to a specific DNA motif was determined by a commercial assay kit (Raybiotech, Norcross, GA). Microtiter plates were coated with double stranded oligonucleotides containing the GATA-3 binding sequence. These oligonucleotides specifically capture the active GATA-3 present in nuclear extracts. Subsequently, the captured GATA-3 was detected by GATA-3 primary antibody and an HRP-conjugated secondary antibody. Hydrogen peroxide/tetramethylbenzidine substrate was used to develop the color which was proportional to bound HRP activity. The reaction was stopped, and the optical density of the color was read at 450 nm in a plate reader (FLUOstar). The specificity of the reaction between active GATA-3 and the DNA probe was additionally tested by specific competitive DNA and non-specific competitive DNA probes.

### IWP-2 inhibition of Wnt processing

Prostate stromal cells (PSC) were treated with the porcupine inhibitor IWP-2 (N-(6-Methyl-2-benzothiazolyl)-2-[(3,4,6,7-tetrahydro-4-oxo-3-phenylthieno[3,2-d] pyrimidin-2-yl) thio]-acetamide; Sigma; 1 μg/ml × 24h), which inhibits Wnt palmitoylation and impairs Wnt secretion. Prostate epithelial cells and HepG2 cells were treated with this spent media in 1:1 ratio with epithelial cell media following control silencing and ARSB silencing. CHST15 expression and phospho-p38/total p38 MAPK were determined following exposure.

### Inhibition of Rho, Rac-1, and β-catenin activation

Prostate epithelial cells (PEC) were treated with JW67, an inhibitor of Wnt/β-catenin signaling by inducing β-catenin destruction (4 mg/ml, Tocris, Bio-Techne, Minneapolis, MN); Rho inhibitor Y-27632 (10 μM, Calbiochem, Millipore/Sigma, Danvers, MA), and Rac-1 inhibitor (50 μM; NSC-23766 (*N*6-[2-[[4-(diethylamino)-1-methylbutyl] amino]-6-methyl-4-pyrimidinyl]-2-methyl-4,6-uinolinediamine trihydrochloride; Tocris, Bio-Techne) for 24 h following control silencing and ARSB silencing. CHST15 expression and phospho-p38/total p38 MAPK were determined as stated above. The effect of NSC-23766 was also detected in HepG2 and PC3 cell lines.

### Neutralization of DKK3 secreted from prostate epithelial cells

Secreted DKK3 from prostate epithelial cells (PEC) following control and ARSB silencing, was captured by DKK3 antibody (0.2 μg/ml, AF1118; R&D System). Control cells were treated with non-specific IgG and treated with prostate stromal cell spent media (SCM) 1:1 with epithelial cell media. CHST15 expression and phospho-p38 MAPK/total p38 MAPK were determined, as above.

### Isolation, purification or fractionation of GAGs for disaccharide analysis

Isolation of GAGs from samples was described previously [53]. The samples were individually subjected to proteolysis at 55° C with 10% (w/v) of actinase E (20 mg/ml in HPLC grade water, Kaken Biochemicals, Tokyo, Japan) for 20 h. After proteolysis, particulates were removed from the resulting solutions by passing each through a 0.22-μm membrane syringe filter. Samples were then concentrated using Microcon YM-10 centrifugal filter units (10 kDa molecular weight cutoff, Millipore) by centrifugation at 12,000 × g and washed with 15 ml of distilled water to remove peptides. The retentate was collected and lyophilized, and then purified or fractionized.

Samples were dissolved in 0.5 ml of 8 M urea containing 2% CHAPS (pH 8.3). A Vivapure Mini Q H spin column (Viva science, Edgewood, NJ) was prepared by equilibrating with 200 μl of 8 M urea containing 2% CHAPS (pH 8.3). To remove any remaining proteins, the clarified, filtered samples were loaded onto and run through the equilibrated Vivapure Mini Q H spin columns under centrifugal force (700 × g). The columns were then washed with 200 μl of 8 M urea containing 2% CHAPS at pH 8.3, followed by five washes with 200 μl of 200 mM NaCl. The total GAGs were released from the spin column by washing three-times with 200 μl of 2.0 M NaCl. For fractionation, the column was eluted by washing 3-times with 200 μl of 0.5, 1.0 and 2.0 M NaCl solution, respectively. The GAGs were collected, desalted using YM-10 spin columns and finally lyophilized.

### Enzymatic digestion of GAGs

The recovered GAGs were next completely depolymerized using polysaccharide lyases. Polysaccharide lyases cleave the glycosidic linkages between hexosamine and uronic acid residues in glycosaminoglycans, converting these polysaccharides into disaccharides containing unsaturated uronic acid residues (ΔUA) at their non-reducing ends. Chondroitin lyase ABC (5 m-units, Seikagaku, Japan) and chondroitin lyase ACII (2 m-units, Seikagaku) in 10 μl of 0.1% BSA were added to a ~5 μg GAG sample in 25 μl 50 mM Tris solution containing 60 mM sodium acetate at pH 8.0, and incubated at 37° C for 10 h. The enzymatic products were recovered by centrifugal filtration at 12,000 × g. CS/DS disaccharides that passed through the filter were freeze-dried for LC-MS analysis. GAGs remaining in the retentate were collected by reversing the filter and spinning at 12,000 × g, followed by incubation with 10 m-units of heparin lyase I, II, and III (Seikagaku) at 35° C for 10 h. The products were recovered by centrifugal filtration using a YM-10 spin column, and the disaccharides were collected in the flow-through and freeze-dried.

### Derivatization of unsaturated disaccharides with AMAC

The freeze-dried biological samples containing GAG-derived disaccharides (~5 μg) or a mixture of 8 HS/HP disaccharide standards or 9 CS/DS disaccharide standards (5 μg/ per each disaccharide) was added to 10 μl of 0.1 M AMAC solution (Sigma) in acetic acid (AcOH)/dimethyl sulfoxide (DMSO) (3:17, v/v) and mixed by vortexing for 5 min. Next, 10 μl of 1 M NaBH_3_CN was added to the reaction mixture and incubated at 45° C for 4 h. Finally, the AMAC-tagged disaccharide mixtures were diluted to various concentrations (0.5–100 ng) using 50% (v/v) aqueous DMSO, and LC-MS analysis was performed.

### LC-MS disaccharide composition analysis of CS/DS

LC-MS analyses were performed on an Agilent 1200 LC/MSD instrument (Agilent Technologies, Inc. Wilmington, DE) equipped with a 6300 ion-trap and a binary pump. The column used was a Poroshell 120 C18 column (3.0 × 150 mm, 2.7 μm, Agilent, USA) at 45° C. Eluent A was 80 mM ammonium acetate solution and eluent B was methanol. Solution A and 15% solution B flowed (150 μl/min) through the column for 5 min followed by a linear gradient from 15–30% solution B for 5 to 30 min. The column effluent entered the ESI-MS source for continuous detection by MS. The electrospray interface was set in negative ionization mode with a skimmer potential of –40.0 V, a capillary exit of –40.0 V, and a source temperature of 350° C, to obtain the maximum abundance of the ions in a full-scan spectrum (150–1200 Da). Nitrogen (8 l/min, 40 psi) was used as a drying and nebulizing gas. Quantification analysis of AMAC-labeled disaccharides was performed using calibration curves constructed by separation of increasing amounts of unsaturated disaccharide standards (0.1, 0.5, 1, 5, 10, 20, 50, 100 ng/each). Linearity was assessed based on amount of disaccharide and peak intensity in extract ion chromatography (EIC).

Standards of CS/DS (ΔDi-0S: ΔUA-GalNAc, ΔDi-4S: ΔUA-GalNAc4S, ΔDi-6S: ΔUA-GalNAc6S, ΔDi-2S: ΔUA2S-GalNAc, ΔDi-diSB: ΔUA2S-GalNAc4S, ΔDi-diSD: ΔUA2S-GalNAc6S, ΔDi-diSE: ΔUA-GalNAc4S6S, ΔDi-triS: ΔUA2SGal-NAc4S6S) and of HA (ΔDi-UA-GlcNAc) were obtained from Seikagaku Corporation (Japan). The molecular weights (MW) of the CS disaccharides are: Di-0S (UA-GalNAc) 379; Di-4S (UA-GalNAc4S) 459; Di-6S (UAGalNAc6S) 459; Di-2S (UA2S-GalNAc) 459; Di-diSB (UA2S-GalNAc4S) 539; Di-diSD (UA2S-GalNAc6S) 539; Di-diSE (UA-GalNAc4S6S) 539; and Di-triS (UA2SGalNAc4S6S) 637.

### Statistical analysis

Data presented are the mean ± SD of at least three independent experiments. Mean values for each sample were calculated as the average of two technical duplicates of each measurement. Statistical significance was determined by one-way analysis of variance followed by post-hoc Tukey’s test for multiple determinations using InStat3 software (GraphPad, La Jolla, CA), unless stated otherwise. Unpaired *t*-tests, two-tailed, were used for two-way comparisons. *P* values ≤ 0.05 are considered to be statistically significant and are indicated by ^*^ or ^#^ for *p* < 0.05, ^**^ or ^##^ for *p* ≤ 0.01, and ^***^ or ^###^ for *p* ≤ 0.001 in comparison to control.
